# Crop breeding by design: integrating big data for future food security

**DOI:** 10.1093/nsr/nwag288

**Published:** 2026-05-18

**Authors:** Shizhong Xu

**Affiliations:** Department of Botany and Plant Sciences, University of California, Riverside, USA

The global agricultural landscape stands at a critical juncture. As the world’s population continues to grow and climate change introduces increasingly unpredictable stressors, the traditional paradigms of crop improvement must evolve. The transition from conventional phenotypic selection to a high-precision, data-driven ‘breeding by design’ (BBD) approach is no longer just a theoretical goal—it is becoming a biological and statistical reality. Drawing from a series of recent breakthroughs in genomics, epigenetics and algorithmic development, we can now see a comprehensive molecular roadmap for the next generation of crops. These advancements, ranging from new statistical frameworks for non-normal traits to the discovery of epigenetic switches for yield and resilience, collectively herald a new era of agricultural productivity and nutritional security.

As the heart of modern breeding is the pyramiding of favorable genes that could be efficiently identified with the assist of the genome-wide association studies (GWAS), yet traditional GWAS models have often struggled with the sheer volume of data generated by large-scale populations and multi-environmental trials. Two pivotal articles in this special issue address these computational and statistical bottlenecks. The first, introducing the Fast3VmrMLM algorithm that includes all possible genetic effects and controls all possible polygenic backgrounds [1], presents a transformative leap in our ability to analyze gene-by-environment interactions (GEIs). Identifying GEIs is crucial for understanding how cultivars adapt to climate change, but the computational cost of analyzing large samples across numerous environments has historically been prohibitive. By integrating eight advanced big-data techniques—such as preconditioned conjugate gradients and vectorized Wald tests—Fast3VmrMLM allows researchers to process datasets comprising tens of thousands of individuals and millions of markers in a fraction of the time required by previous methods. This algorithm does more than just find genes; it deciphers phenotypic plasticity by GEIs identified from the regression parameters of phenotypes on meteorological factors like photothermal time and photosynthetically active radiation. In maize, for instance, this tool identified key regulators that explain why certain hybrids flower earlier or later depending on their environment, providing a direct mechanism for breeders to design climate-adaptive cultivars. Furthermore, Wang *et al*. extended traditional SNP-based GWAS to a Pan-marker GWAS framework, including bin haplotypes and other molecular variants, thereby improving the efficiency of identifying rare-variants.

Parallel to this, the development of the PSR-GLMM (Pseudo Response Generalized Linear Mixed Model) framework addresses a different but equally critical challenge: the analysis of traits that do not follow a normal distribution [2]. Many important agronomic traits are binary (e.g. disease presence), count-based (e.g. lesion spots) or ordinal (e.g. disease severity scores). Treating these as continuous variables often leads to biased results and a failure to control for ‘Type 1’ errors. The PSR-GLMM method generates a pseudo-response variable that allows these discrete traits to be analyzed with the statistical rigor of a linear mixed model while maintaining high computational efficiency. Through validations in rice, Arabidopsis, pigs and dogs, PSR-GLMM has proven to be an essential tool for unlocking the genetic architecture of complex, non-linear traits that are vital to both animal and plant health.

Beyond the tools of detection, we must understand the ‘how’ and ‘why’ of crop evolution. Recent integrative multi-omics analyses have provided unprecedented insights into the domestication of specialty crops, most notably black rice [3]. Long prized for its nutritional superiority, black rice was found to have a unique two-step domestication trajectory. Contrary to earlier assumptions, genomic evidence suggests that black rice originated not directly from wild ancestors, but as a ‘post-domestication improvement’ from white rice. This transition was driven by a gain-of-function mutation in the *OsKala4* gene, which activated anthocyanin biosynthesis in the pericarp. Furthermore, the identification of previously uncharacterized fine tuners like *OsWRKY1* and *OsBBX13* has revealed a synergistic regulatory network that enhances the core MBW transcriptional complex (MYB, bHLH, WD40) responsible for pigmentation. This discovery is vital for breeders because anthocyanin biosynthesis often comes with a ‘yield penalty’ due to metabolic resource competition and reduced photosynthetic efficiency in vegetative tissues. By identifying these specific regulators, scientists can now pursue ‘precision-breeding’ strategies that decouple nutritional enrichment from agronomic trade-offs, effectively designing ‘green nutritious super rice’ that is both high-yielding and nutrient-dense.

Similarly, the study of the SAG9 (seed-aging gene 9) locus provides a cautionary tale of what can be lost during domestication [4]. High-quality seeds require a coordination between dormancy (to prevent pre-harvest sprouting) and storability (to preserve vigor). *SAG9* was identified as a negative regulator of both traits. Interestingly, an elite haplotype of *SAG9* (*Hap-*) that enhances storability and dormancy has been largely lost in modern varieties, particularly those in southern China. This loss occurred because domestication favored ‘strong vigor’—seeds that germinate rapidly and uniformly—which often creates a trade-off with long-term survival. By reintroducing these ‘lost’ alleles through gene editing or marker-assisted selection, breeders can restore seed longevity in modern high-yield cultivars, ensuring they remain viable in the high-temperature and high-humidity environments of the tropics.

The final pieces of the BBD puzzle involve direct molecular interventions to enhance yield and stress tolerance [5]. One of the most promising strategies involves the epitranscriptome—specifically RNA m^5^C (5-methylcytosine) methylation. While DNA methylation is well-studied, RNA methylation has only recently emerged as a key regulator of plant productivity. Research into the RNA m^5^C demethylase *OsNOP2* has shown that ‘erasing the eraser’ can lead to massive yield gains. Knocking out *OsNOP2* leads to elevated RNA m^5^C levels, which, in turn, increases the translation efficiency of transcripts involved in carbon assimilation and nitrogen metabolism. In field trials, *OsNOP2-KO* rice plants exhibited a 28% increase in grain yield per plot. Crucially, this yield boost was maintained under abiotic stressors like heat and high salinity. Perhaps most importantly, this mechanism appears to be functionally conserved; knocking out NOP2 orthologs in wheat and tomato resulted in similar productivity gains, suggesting that targeting RNA methylation is a universal strategy for increasing global crop yields.

In the realm of resilience, the discovery of the *TaWRKY115* gene in wheat offers a sophisticated model for enhancing cold tolerance [6]. Wheat is a staple for billions, yet low-temperature stress causes significant annual yield losses. Researchers identified *TaWRKY115* as a positive regulator that enhances cold tolerance through a specific molecular cascade: *TaSP1-TaWRKY115-TaMYB4-TaCBF*. Under cold stress, *TaWRKY115* acts as a ‘dual suppressor’ of *TaMYB4*, inhibiting it at both the transcriptional and protein levels. This releases the inhibition of *TaCBF* family genes, which then activate cold-responsive (COR) pathways to protect the plant. The identification of superior haplotypes of *TaWRKY115*—which are present in some landraces but not widely utilized in modern breeding—provides a clear target for improving the winter hardiness of elite wheat varieties.

The collective findings from these six articles represent more than just isolated discoveries; they are the building blocks of a unified agricultural strategy. We now possess the statistical tools (Fast3VmrMLM and PSR-GLMM) to handle massive datasets and non-normal traits. We have the historical context (Black Rice and SAG9) to understand the trade-offs between nutrition, vigor and domestication. And we have the molecular switches (RNA m5C and WRKY-cascades) to directly boost yield and environmental resilience. The future of agriculture lies in the integration of these insights. ‘Breeding by design’ is no longer about selecting the best-looking plant in the field. It is about using high-throughput algorithms to predict how a specific gene haplotype will interact with next year’s predicted solar radiation. It is about engineering the epitranscriptome to maximize nitrogen-use efficiency while simultaneously reintroducing ancient alleles to ensure those seeds survive storage. As we move toward a ‘healthy Anthropocene’, these advancements offer the precision needed to develop crops that are not only productive and resilient but also nutritionally optimized for the human diet. The molecular roadmap is clear; the task now is to build the crops it describes.
